# New Insights into Pathophysiology of Vestibular Migraine

**DOI:** 10.3389/fneur.2015.00012

**Published:** 2015-02-06

**Authors:** Juan M. Espinosa-Sanchez, Jose A. Lopez-Escamez

**Affiliations:** ^1^Otology and Neurotology Group CTS495, Human DNA Variability Department, GENYO Centre for Genomics and Oncological Research Pfizer – University of Granada – Junta de Andalucia, Granada, Spain; ^2^Department of Otolaryngology, Hospital San Agustin, Linares, Spain; ^3^Department of Otolaryngology, Hospital de Poniente, El Ejido, Spain

**Keywords:** migraine, aura, vertigo, multisensory integration, vestibulo-thalamo-cortical system, Meniere’s disease, vestibular system

## Abstract

Vestibular migraine (VM) is a common disorder in which genetic, epigenetic, and environmental factors probably contribute to its development. The pathophysiology of VM is unknown; nevertheless in the last few years, several studies are contributing to understand the neurophysiological pathways involved in VM. The current hypotheses are mostly based on the knowledge of migraine itself. The evidence of trigeminal innervation of the labyrinth vessels and the localization of vasoactive neuropeptides in the perivascular afferent terminals of these trigeminal fibers support the involvement of the trigemino-vascular system. The neurogenic inflammation triggered by activation of the trigeminal-vestibulocochlear reflex, with the subsequent inner ear plasma protein extravasation and the release of inflammatory mediators, can contribute to a sustained activation and sensitization of the trigeminal primary afferent neurons explaining VM symptoms. The reciprocal connections between brainstem vestibular nuclei and the structures that modulate trigeminal nociceptive inputs (rostral ventromedial medulla, ventrolateral periaqueductal gray, locus coeruleus, and nucleus raphe magnus) are critical to understand the pathophysiology of VM. Although cortical spreading depression can affect cortical areas involved in processing vestibular information, functional neuroimaging techniques suggest a dysmodulation in the multimodal sensory integration and processing of vestibular and nociceptive information, resulting from a vestibulo-thalamo-cortical dysfunction, as the pathogenic mechanism underlying VM. The elevated prevalence of VM suggests that multiple functional variants may confer a genetic susceptibility leading to a dysregulation of excitatory–inhibitory balance in brain structures involved in the processing of sensory information, vestibular inputs, and pain. The interactions among several functional and structural neural networks could explain the pathogenic mechanisms of VM.

Vestibular migraine (VM) is the most common cause of spontaneous episodic vestibular syndrome with a lifetime prevalence of about 1% (1). VM occurs in patients with current or previous history of migraine and recurrent episodes of vestibular symptoms accompanied by migraine features. In 2001, Neuhauser et al. proposed early diagnostic criteria for VM (2), and they have proven a high reliability in longitudinal studies (3); these criteria included:

1. Episodic vestibular symptoms of at least moderate intensity.
2. Migraine according to the International Headache Society (IHS).
3. At least one of the following migrainous symptoms in at least two vertiginous attacks: migrainous headaches, photophobia, phonophobia, and visual or other auras.
4. Other causes ruled out by appropriate investigations.

Currently, the diagnosis is based on the criteria developed by the Bárány Society and the IHS (4). The major differences between Neuhauser and Barany Society-IHS criteria are that the new definition sets out at least five episodes of vestibular symptoms with a duration of 5 min – 72 h. Furthermore, current diagnostic criteria use the Bárány Society’s Classification of Vestibular Symptoms (5).

The dissemination of these criteria and the inclusion in the beta version of the 3rd edition of the ICHD has renewed the interest in VM (6–9).

Neuro-otological examination between episodes is usually normal, although spontaneous and positional nystagmus can be found during the attacks. Vestibular function tests reveal normal findings in almost half of the patients, even though unilateral vestibular hypofunction and oculo-motor disturbances are also common.

The differential diagnosis includes benign paroxysmal positional vertigo (BPPV), Meniere’s disease (MD), labyrinth transient ischemic attacks, superior canal dehiscence syndrome, and chronic subjective dizziness, being the distinction between MD and VM the major diagnostic challenge, particularly in the first years of the disease.

The pathophysiology of VM is unknown. Several hypotheses have been proposed to explain the concurrence of episodic vestibular symptoms and migraine. Vestibular symptoms may appear in any phase of a migraine attack with a variable duration. In some episodes, there are only migraine symptoms, while in others migraine and vestibular symptoms are observed. Interestingly, within a single family, some members may suffer from migraine and other relatives have all the symptoms of VM. Moreover, it is unclear the relation between VM, MD, and BPPV (10).

We summarize the current knowledge on the pathophysiology of VM with a particular focus on genetic studies and multisensory integration network. Firstly, we will describe the mechanisms of migraine itself, since current hypotheses are mostly based on its pathophysiology.

## Pathophysiology of Migraine

Migraine is a complex disorder with a controversial pathophysiology. In the last few years, several investigations have contributed to elucidate the causal mechanisms. It is widely accepted that the trigemino-vascular system (TVS) is the anatomical substrate and its activation and sensitization causes headache. The electrophysiological phenomenon of cortical spreading depression (CSD) could be the neurophysiological correlate of migraine aura, although CSD has not been linked with aura in humans (11).

The TVS involves the nociceptive pseudounipolar neurons of the trigeminal ganglion (TG), which innervate the pia, the dura mater, and the cranial blood vessels, mainly through the ophthalmic division of the trigeminal nerve (12). These first-order neurons project centrally to the trigemino-cervical complex (TCC), including the caudal portion of the trigeminal spinal nucleus (trigeminal nucleus caudalis) and the C_1_ and C_2_ dorsal horns of the spinal cord. The projections of the second-order neurons of TCC ascend through the quintothalamic tract and connect to the contralateral ventroposteromedial (VPM) and posterior thalamic nuclei, which in turn project to the primary and secondary somatosensory cortex, the insular cortex, and the anterior cingulate cortex. Moreover, the TCC makes reciprocal connections to brainstem centers involved in the processing of nociceptive information. These centers include the rostral ventromedial medulla (RVM) – particularly the nucleus raphe magnus – the ventrolateral periaqueductal gray (vlPAG), and hypothalamic areas. In fact, the modulation of the TCC depends upon descending pathways from the cortex, and posterior hypothalamus and these brainstem nuclei (13).

Experimental studies have demonstrated that the depolarization of the peripheral terminals of meningeal nociceptors leads to the activation of the TVS with the release of vasoactive neuropeptides from their perivascular endings. These substances, mainly substance P (SP), calcitonin gene-related peptide (CGRP), and neurokinin A (NK-A), cause vasodilation and an increase in cerebral blood flow, with plasma protein extravasation and mast cell degranulation on the dura with the release of pro-inflammatory factors that finally result in an inflammatory reaction known as neurogenic inflammation (11).

Pro-inflammatory mediators and the released glutamate increase the excitability of the first-order neurons in the TG (peripheral sensitization) producing throbbing pain. These mediators also sensitize second-order and third-order trigemino-vascular neurons (central sensitization) causing allodynia (14–16). Functional brain imaging studies show a participation of vlPAG, RVM, and nucleus cuneiformis in the maintenance of central sensitization.

Cortical spreading depression consists of a transient but massive neural depolarization, which generates a wave that slowly spread across cortex from occipital area, and it is followed by a prolonged suppression of cortical bioelectrical activity. CSD causes disruptions of transmembrane ionic gradients, increasing the intracellular concentrations of Ca^2+^ and the extracellular concentrations of H^+^, K^+^, glutamate, arachidonic acid, and nitric oxide (NO) in the synaptic cleft. These molecules are able to activate perivascular and meningeal nociceptors of TVS (17) and also central trigemino-vascular neurons in the spinal trigeminal nucleus (18).

The initiation of CSD is ignited by a local increase in the extracellular K^+^ concentration above a critical value and the release of glutamate from apical dendrites of cortical pyramidal cells and activation of NMDA receptors, probably due to the opening of voltage-gated Ca^2+^ channels (19). Recently, it has been experimentally demonstrated that CSD induces opening of neuronal pannexins channels and the release of pro-inflammatory substances that may activate the meningeal TVS (20).

Moreover, the concept of CSD as a migraine trigger is controversial since aura is not present in most patients with migraine. Some authors have postulated a silent aura mechanism, but the initial mechanism that causes activation of TVS is not known. A recent hypothesis proposes that migraine is a pathological brain state (21), in which multiple partially overlapping brain networks are involved. So, migraine headache arises from a dysfunction within diencephalic structures and brainstem nuclei that modulate trigeminal nociceptive inputs: RVM, vlPAG, posterior hypothalamus, and VPM thalamic nucleus (13). In this alternative hypothesis, direct activation of the TVS would occur instead of through activation of peripheral nociceptors and sequential sensitization of the first-, second-, and the third-order trigemino-vascular neurons.

## Ischemic Events and VM

The hypotheses to explain the pathophysiology of VM have evolved over time from the vascular to the neurogenic theory. Reversible vasospasm of the internal auditory artery or its branches was one of the first proposed explanations (22). This mechanism could be responsible for the sudden onset of vestibular and/or auditory symptoms, spontaneous nystagmus or even could account for the link between VM and MD or BPPV. Moreover, a population-based study indicates that migraine could be associated with sudden sensorineural hearing loss (23).

Central spontaneous nystagmus and ocular-motor abnormalities cannot be elucidated by internal auditory artery ischemia, but it may be the manifestation of infarction in the anterior inferior cerebellar artery territory (AICA) (24). Meta-analyses indicate that migraine and stroke are not only comorbid conditions (25), but rather migraine is probably an independent cerebrovascular risk factor, especially in young people with migraine with aura (26, 27).

Several studies have reported reduced or absent vestibular evoked myogenic potential (VEMP) responses in patients with VM indicating a dysfunction of the vestibulo-collic reflex (28–31). These findings could be related with the hypoperfusion of the inner ear affecting the otolith organs, as well as ischemic lesions of the descending otolith pathways in the brainstem.

## Cortical Spreading Depression and VM

Although the vestibular symptoms in VM should not be consider as a type of sensory aura, it has been postulated that they could be explained by the mechanism of aura, i.e., CSD (32). The wave of CSD could reach the so-called vestibular cortex or even the brainstem vestibular nuclei causing vestibular symptoms. However, the duration of VM episodes (from seconds to days) does not fulfill the characteristics of an aura, and neither explains the unilateral canal paresis found in some patients. Aura can occur at any time during the attack, and aura without headache can also be observed. Furthermore, in the case of migraine with brainstem aura, previously called basilar-type, there are other brainstem symptoms such dysarthria, tinnitus, hypacusis, diplopia, ataxia, or decreased level of consciousness.

## Involvement of TVS in VM

The inner ear receives innervation from the ophthalmic branch of the TG through the basilar artery and the AICA (33). The TG also innervates the cochlear nucleus and the superior olivary complex (34). In addition, experimental studies have shown that chemical and electrical stimulation of the TG cause a significant increase in the inner ear blood flow, and changes in vascular permeability with plasma protein extravasation into the inner ear (35–37). These findings open the doors to the participation of the TVS in the pathophysiology of VM. In this sense, painful trigeminal electric stimulation in patients with migraine without aura prompts a spontaneous nystagmus of probable peripheral origin, or modifies pre-existing spontaneous nystagmus, mostly increasing it (38). It has been identified the presence of vasoactive neuropeptides (e.g., SP, CGRP) in the trigeminal sensory fibers innervating the inner ear and in the vestibular nuclei (36, 39).

## Genetic Factors

Recently, several genome-wide association studies (GWAS) have identified common genetic variants at several loci significantly associated with migraine susceptibility (40, 41). Three markers have shown a consistent association in independent cohorts of patients with migraine: rs2651899 in the *PRDM16* gene, rs10166942 in the*TRPM8* gene, and rs11172113 in the *LRP1* gene. The common variant in the *PRDM16* gene has been associated with migraine in European, Chinese, and North Indian populations, but the role of this gene in migraine is unknown.

Mutations in *CACNA1A* gene, which encodes the central pore-forming subunit of the voltage-gated Ca_V_2.1 (P/Q-type) calcium channels, cause at least three neurological calcium channelopathy syndromes: episodic ataxia type 2, familial hemiplegic migraine type 1, and spinocerebellar ataxia type 6 (42). Accumulating evidence suggests that Ca_V_2.1 channels could be involved in migraine and VM pathogenesis (6, 43). Thus, migraine may be conceived as a channelopathy in which CSD would be the result of different mutations that would increase the susceptibility to CSD by affecting several ionic channels, mostly involved in glutamate homeostasis, such that the common final result would be an increase of glutamate and K^+^ extracellular concentrations in the synaptic cleft, which could contribute to TVS activation, central sensitization, and CSD initiation (44).

Familial occurrence of VM has been reported suggesting a genetic component. Within the same family, there can be multiple-affected relatives, some individuals may have migraine and others VM or benign paroxysmal vertigo of childhood, suggesting a phenotypic heterogeneity. An autosomal dominant pattern with moderate to high penetrance is frequently appreciated (45, 46). However, Sanger sequencing did not identified mutations in patients with VM in genes causing familial hemiplegic migraine, such as *CACNA1A*, *ATP1A2*, *SCN1A*, or episodic ataxia type 5 (*CACNB4*) (45, 46).

Despite no causative mutation has been found in candidate gene studies, several loci have been identified. Lee et al. (47) studied 20 multicase families diagnosed with benign recurrent vertigo (BRV), demonstrating linkage to 22q12. BRV includes patients with recurrent episodes of spontaneous vertigo in the absence of cochlear signs (48). Nowadays, most of the patients previously diagnosed with BRV could be classified as VM, since 79% of the individuals diagnosed with BRV also fulfill criteria of migraine (47).

Subsequently, the same authors studied a large pedigree with VM (49). These investigators did not find a region of the genome shared by all affected family members, indicating a polygenic inheritance. Nevertheless, a region on 11q was shared by most affected females, suggesting that may contain a susceptibility allele that is penetrant predominantly in women.

Bahmad et al. studied a family with 10 members affected by VM demonstrating an autosomal dominant trait. Genome-wide linkage analysis and subsequent fine mapping revealed that the disease gene is located between markers rs244895 and D5S2073 in chromosome 5q35 (50).

According to prevalence of VM, it is unlikely to be a monogenic disease as familiar hemiplegic migraine or episodic ataxias, but it would be rather polygenic as MD. The involved functional genetic variants of multiple ionic channels and receptors, both in vestibular pathways and in the pain network, may determine the observed clinical heterogeneity in the VM phenotype. This would explain the concurrence of peripheral and central vestibular findings, and the migrainous features.

## Multisensory Integration and Vestibulo-Thalamic-Cortical Processing

The pathophysiology of VM has been explained by interactions between nociceptive and vestibular pathways (51). The reciprocal connections between the vestibular nuclei and other brainstem structures, such as the parabrachial nucleus, the raphe nuclei, and the locus coeruleus, may contribute to modulate the sensitivity of trigemino-vascular reflex and pain pathways (52–54). Positron emission tomography (PET) and functional magnetic resonance imaging (fMRI) have revealed an activation of the dorsal brainstem during migraine attacks, significantly at the dorsal raphe nucleus and the vlPAG. In addition, reciprocal connections between the trigeminal nucleus caudalis and the vestibular nuclei could explain vestibular symptoms in migraneurs.

Some studies showed that patients with VM may have enhanced perceptual sensitivity for head motions that dynamically modulate canal and otolith inputs together (55), so it has been suggested that canal–otolith integration may be abnormal in these patients due to an altered signal processing in the caudal cerebellar vermis (56).

Electrophysiological and neuroanatomic studies in animals, along with functional neuroimaging techniques in humans by PET and fMRI, have identified the vestibular thalamus and the vestibular cortex as an essential part of the central vestibular system (57). Multiple thalamic nuclei are involved in vestibular processing, particularly the ventralposterolateral (VPL) and VPM nuclei. The thalamus is a major sensory relay station in the vestibular pathways, and it participates in the multisensory integration and processing of vestibular, visual, and proprioceptive inputs, so thalamic function could be a key event in multimodal sensorial sensitization. Thalamic activation has been demonstrated during a migraine attack using fMRI. VPL has been involved in controlling body orientation in space and the VPM receives trigeminothalamic input. We speculate that migraine attacks may transiently sensitize VPL and VPM nuclei and enhance the sensory perception, including vestibular information. This hypothesis would also explain the susceptibility to motion sickness described in migraine and VM patients (58, 59).

Although a specific vestibular cortex has not been clearly identified in humans, several cortical areas are involved in the processing of vestibular information, particularly the insula, the parietal operculum, and the temporo-parietal junction, as well as the posterior parietal cortex, the cingulate cortex, the somatosensory cortex, and the lateral and medial frontal cortices (60–63). These regions are multisensory areas where several sensory modalities converge. Equally, a nociceptive-specific area neither has been found, but it is worth noting that the posterior insular–opercular region has been postulated as the primary nociceptive cortex (64) and it also may provide a site for interaction between vestibular and nociceptive processing (61, 65). The vestibular cortical projections strongly overlap with the somatosensory cortical projections, probably due to intrainsular connections, giving rise to vestibular–somatosensory interactions as proven by both caloric and galvanic vestibular stimulation (66, 67). So, it has been suggested that vestibular input could influence processing in other sensory modalities.

This thalamo-cortical involvement has also been demonstrated in PET studies in VM (68). Subtraction images comparing ictal with interictal images showed an increased metabolism in the bilateral cerebellum, frontal cortices, temporal cortex, posterior insula, and thalami. It was also observed hypometabolism in the bilateral occipitotemporal and posterior parietal cortices. The cerebellar hypermetabolism is interpreted by an adaptive mechanism to suppress the hyperactive vestibular system, whereas the occipitotemporal deactivation is explained by reciprocal inhibition between visual and vestibular systems (69). This study indicates activation of the vestibulo-thalamo-cortical pathway in VM.

Neuroimaging in migraineurs with blood oxygenation level-dependent (BOLD) fMRI, have revealed the participation of several cortical areas others than primary visual cortex. These include anterior cingulate, prefrontal, and orbitofrontal cortices. Using BOLD fMRI, caloric vestibular stimulation elicited a significant activation in insular cortex, parietal cortex, thalamus, brainstem (including PAG), and cerebellum, and a significantly decreased response in anterior cingulate cortex. This pattern is observed in both healthy volunteers and patients with migraine without aura or VM during an interictal phase (70). Nevertheless, patients with VM showed also an increased mediodorsal thalamic activation ipsilateral to the vestibular stimulation as compared with both, healthy controls and patients with migraine without aura. Moreover, the degree of thalamic activation was correlated with frequency of migraine attacks in patients with VM.

Recently, patients with VM have been studied using MRI-based voxel-based morphometry to analyze gray matter (GM) volume differences between VM patients and healthy controls (71). VM patients showed a decrease of GM volume bilaterally in the inferior temporal gyrus, the cingulate cortex, and the posterior insula. GM decrease was also observed in the left side of superior temporal gyrus, middle temporal gyrus, supramarginal gyrus, and superior parietal lobules, and on the right sides of the dorsolateral prefrontal cortex and the inferior occipital gyrus. These findings are consistent with above-mentioned studies that suggested that these areas are involved in cortical processing of vestibular and nociceptive information.

An intrinsic hypersensitivity to different sensory stimuli has been described in migraineurs. Exposure to a specific stimulus results not only in hypersensitivity to that stimulus, but it may also result in further enhancement of hypersensitivity to other stimuli. It has been appreciated an altered central processing of sensory stimuli in patients with migraine including trigeminal inputs (72), in such a way that some authors interpret migraine as a disorder of multisensory integration (73). As we have seen, multisensory integration has been described in all vestibular relays, including vestibular nuclei, thalamus, and cerebral cortex. These multimodal sensory areas are responsible for the integration and processing of somatosensory, visual, and vestibular information. An intrinsic enhanced vestibular sensory perception and these cross-modal interactions at (vestibular) brainstem-thalamo-cortical level could explain, at least partly, the link between migraine and some vestibular disorders including VM.

**Figure 1:**
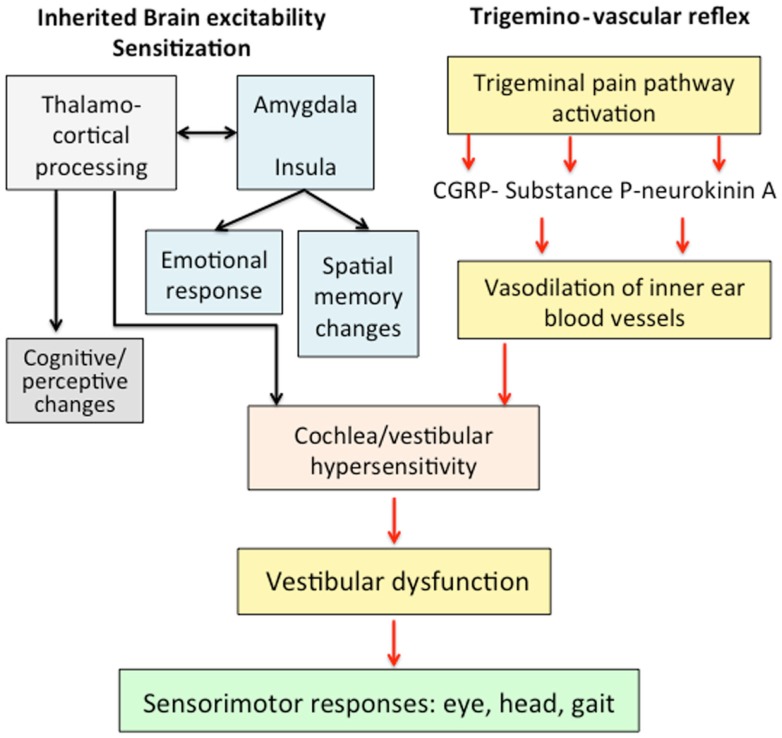
**Mechanisms involved in the pathophysiology of vestibular migraine**. An abnormal brain sensitization leading to a dysmodulation of multimodal sensory integration in thalamo-cortical processing could interact with the trigemino-vascular reflex. The abnormal processing of vestibular and nociceptive information could determine a transient vestibular dysfunction associated with migraine features.

We hypothesized that VM responds to an abnormal brain sensitization leading to a dysmodulation of multimodal sensory integration and processing at vestibulo-thalamo-cortical level (Figure 1). At the molecular level, this may result from functional variants affecting several ionic channels and receptors, and that can confer susceptibility to VM. Multiple brain networks are probably involved not only in the pain matrix and the vestibular pathways, but also in other structures such as the limbic system.

## Conflict of Interest Statement

The authors declare that the research was conducted in the absence of any commercial or financial relationships that could be construed as a potential conflict of interest.
